# Accurate Methane Detection in Combustible Gas Mixtures by Using SnO_2_‑Ag-ZnO Gas Sensors with Rapid Responses

**DOI:** 10.1021/acssensors.5c02966

**Published:** 2025-12-15

**Authors:** Mingzhi Jiao, Haojie Dong, Yuting Qiao, Ruqi Guo, Chu Manh Hung, Nguyen Van Duy, Nguyen Duc Hoa, Chenyu Wen

**Affiliations:** † National and Local Joint Engineering Laboratory of Internet Application Technology on Mine, 12392 China University of Mining and Technology, Xuzhou 221116, China; ‡ School of Information and Control Engineering, China University of Mining and Technology, Xuzhou 221116, China; § International Training Institute of Materials Science, 118018 Hanoi University of Science and Technology, Hanoi 100000, Vietnam; ∥ Division of Solid-State Electronics, Department of Electrical Engineering, Ångströmlaboratoriet, 8097 Uppsala University, Lägerhyddsvägen 1, Uppsala 75237, Sweden

**Keywords:** methane sensor, SnO_2_ composite, heterostructure, SqueezeNet, gas classification

## Abstract

Efficient monitoring of methane is crucial for avoiding gas explosions in industrial processes. Metal oxide methane sensors exhibit promising gas detection performance, which has been well studied recently. However, conventional metal oxide sensors suffer from high operating temperatures, limited selectivity in multigas interference scenarios, and insufficient compatibility between hardware efficiency and algorithmic complexity for real-life applications. Here, we developed methane sensors based on SnO_2_-Ag-ZnO composite materials. Experimental results demonstrate that Ag-doping reduces the optimal operating temperature and enhances the methane response 1.79-fold, compared with that of pure SnO_2_ sensors. Introducing ZnO further amplifies gas adsorption and reaction activity by heterojunction effects. Furthermore, the SqueezeNet transfer learning model was applied to analyze the gas response signals, achieving 91.6% accuracy in the classification task of combustible gas mixtures. This research provides a comprehensive solution for monitoring methane in complex gas mixture environments.

## Graphical abstract

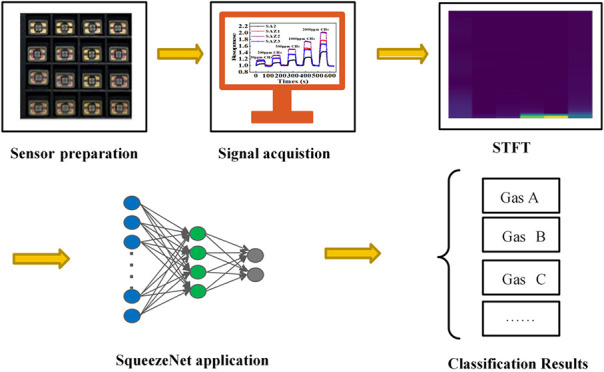

Methane (CH_4_) explosions represent a predominant category of major fire accidents, posing severe threats to personnel security and equipment integrity.^1–4^ Consequently, establishing reliable long-term monitoring systems to detect CH_4_ leakage is imperative for ensuring mining safety and environmental protection. These explosions primarily originate from a mixture of CH_4_ and other combustible gases, including carbon monoxide (CO), hydrogen (H_2_), alkanes, and alkenes.^5^ Research indicates that other combustible gases can enhance CH_4_ reaction sensitivity at even low concentrations, lowering explosion thresholds while amplifying blast intensities.^6,7^ Metal oxide semiconductor (MOS) CH_4_ sensors have garnered significant attention due to their cost-effectiveness, structural simplicity, and potential for miniaturization, facilitating system integration. The nonpolar molecular structure of CH_4_ with sp^3^-hybridized C–H bonds demands a high activation energy (416 kJ/mol) for bond dissociation. Therefore, conventional MOS CH_4_ sensors typically require high operating temperatures and/or external light excitation to overcome the high activation energy barrier for CH_4_ redox reactions and interparticle charge transport limitations.^8–10^ These constraints lead to prohibitively high energy demands, limiting the feasibility of battery-driven Internet of Things (IoT) applications. Microelectro-mechanical system (MEMS) CH_4_ sensors based on microhotplates exhibit low power consumption due to thermal isolation and a small heating area, which facilitates their applications in IoT or portable monitoring systems.

Tin oxide (SnO_2_), a representative n-type MOS with a wide bandgap (3.6 eV), has been extensively utilized in gas sensing due to its high sensitivity, rapid response kinetics, and operational stability.^11–14^ Nevertheless, the practical deployment of SnO_2_ sensors is constrained by high operating temperature and inherent instability under prolonged working conditions.^15–17^ Recent advancements have demonstrated multiple effective strategies for enhancing its CH_4_ sensing performance, including structural optimization,^18^ elemental doping,^19^ and composite formation with other metal oxides,^20^ noble metals,^21^ and 2D materials (*e.g.*, rGO and MoS_2_).^22^ Noble metal doping induces dual enhancement mechanisms through Schottky junction formation at SnO_2_ interfaces and catalytic activation of gas molecule adsorption and reactivity. This combination significantly improves sensitivity and response/recovery kinetics.^23,24^ In 2022, Wang et al. demonstrated that Ag-doped SnO_2_ nanoflowers synthesized via a one-step hydrothermal method achieved a 1.6-fold enhanced response to 500 ppm CH_4_ compared to pristine SnO_2_, attributed to increased oxygen vacancies and catalytic activity.^25^ SnO_2_-based composites with noble metals (e.g., Pd, Pt, and Au) have enhanced gas-sensing properties through catalytic activation and electronic sensitization. Pd-decorated SnO_2_ exhibited a three times higher response to CH_4_ than pristine SnO_2_ by promoting CH_4_ dissociation via spillover effects.^26^ Subsequently, Li et al. reported the fabrication of mulberry-like ZnO/SnO_2_ heterostructures through a two-step hydrothermal synthesis, which exhibited a 56.1% response toward 2000 ppm CH_4_ with a deviation of less than 5% over 30 cycles and selective discrimination against interfering gases.^27^

Despite advancements in SnO_2_-based CH_4_ sensors, cross-sensitivity issues remain unresolved. In complex environments containing multiple reducing gases such as CH_4_, CO, and H_2_, nonspecific sensor responses often lead to misidentification. Support vector machines, random forests, and deep neural networks have been employed to enhance the gas discrimination capability of the MOS gas sensor array.^28^ However, current methods for gas mixture classification often encounter three primary limitations: 1. combinatorial explosion of feature dimensions arising from multicomponent gas interactions, 2. overlapped response patterns caused by cross-sensitive reactions among gas species (e.g., CH_4_, CO, and H_2_), and 3. degraded generalization under dynamic ambient conditions due to nonstationary sensor behavior.

In this work, we developed SnO_2_-Ag-ZnO gas sensors via magnetron sputtering, which enables large-scale fabrication. Comprehensive gas sensing characterization demonstrated that SnO_2_-Ag-ZnO sensors exhibit a superior CH_4_ response compared to pristine SnO_2_ counterparts, with notable improvements in sensitivity and response time. Furthermore, SqueezeNet, a lightweight deep-learning model designed for embedded systems,^29^ was applied to classify CH_4_ mixtures.

## Materials and Methods

### Preparation of Ag-ZnO-SnO_2_ Sensing Layers

A magnetron sputtering system was used for depositing gas-sensitive materials. Key instrumentation specifications, material purity qualifications, and characterization methodologies were summarized in Table S1 in the Supporting Information (SI). Different sensing films were deposited on the silicon microheater substrates (HHC1000, Micro-Nano Sensing Technology Co., China) with the Pt interdigital electrodes to form the sensors.

The sensors were fabricated via the following process. Prepare a 50 mm-diameter, 3 mm-thickness SnO_2_ target (99.99%, Zhongnuo Advanced Material Technology Co., China). Clean target surfaces ultrasonically in ultrapure water to remove particulates, followed by degreasing with 75% ethanol to remove organic contaminants. Then, position targets at 45° angles relative to UV lamps and gas inlets. Mount microheater substrates on rotating platforms using high-temperature-resistant sputtering ceramic holders, and conduct leak detection after the chamber is closed. Initiate vacuum pumping until chamber pressure reaches <5 × 10^–3^ Pa. Introduce argon gas (99.9% purity, Xuzhou Special Gas Plant Co., China) was introduced into the chamber. Adjust the flow rate to achieve a vacuum level of ∼1 Pa. Initiate sputtering and deposit for 1800 s with a power of 50 W. Afterward, switch to Ag target (99.99%, Zhongnuo Advanced Material Technology Co., China) and deposit for 10–30 s with a power of 50 W. Switch to ZnO target (99.99%, Zhongnuo Advanced Material Technology Co., China), and deposit for 100–300 s with a power of 50 W. Finally, anneal the films at 400 °C for 30 min to reduce inner stress between the sputtered layers, thereby reducing the cracks of the films.

Three categories of sensors were fabricated: pure SnO_2_, SnO_2_-Ag composites, and SnO_2_-Ag-ZnO composites. The components and corresponding thicknesses of these sensors are listed in Table 1. It is worth noting that the thickness of each layer in the composite films is a nominal value, interpolated, or extrapolated from calibration curves. A profilometer (Bruker DektakXT system, Bruker Co., Germany) was used to create the calibration curves for film thickness at different deposition times.

**Table 1:** Components of the Sensing Layer

Sample name	SnO_2_ film sputtering time/ nominal thickness	Ag film sputtering time/ nominal thickness	ZnO film sputtering time/ nominal thickness
S0	1800 s/232 nm	-/-	-/-
SA1	1800 s/232 nm	10 s/3 nm	-/-
SA2	1800 s/232 nm	20 s/6 nm	-/-
SA3	1800 s/232 nm	30 s/9 nm	-/-
SAZ1	1800 s/232 nm	20 s/6 nm	100 s/5 nm
SAZ2	1800 s/232 nm	20 s/6 nm	200 s/11 nm
SAZ3	1800 s/232 nm	20 s/6 nm	300 s/16 nm

### Materials Characterization and Sensor Measurement

X-ray photoelectron spectroscopy (XPS, ESCALAB 250Xi, Thermo Fisher Scientific Inc., USA) was employed to analyze the sensing materials, utilizing monochromatic Al Kα radiation (1486.6 eV) as the X-ray source. All spectra were energy-calibrated using the C 1s peak of adventitious carbon at 248.8 eV. Field-emission scanning electron microscopy (FE-SEM, G0AIA3 XMH, TESCAN Group, Czech Republic) was employed to investigate the micromorphology of the sensing materials. Elemental composition and spatial distribution were further analyzed through energy-dispersive spectroscopy (EDS) integrated with a SEM system.

A seven-sensor array was employed to collect data for three pure gases and four mixtures. The total gas volume remained constant across all experiments, with component volume ratios systematically adjusted to achieve the target concentrations. Gas-sensing measurements were conducted under ambient conditions at a room temperature of approximately 26 °C with a relative humidity of approximately 40%. Target gases were introduced into a sealed 5 L chamber housing the sensor array. Sensor resistance values, captured via a measurement board, were transmitted to a computer for analysis and processing. During the measurement, each gas concentration exposure consisted of two 200 s phases: gas injection and purging (total of 400 s). The sensors were biased at a constant voltage of 2 V, and the corresponding current was recorded at 1 Hz using a 9064C instrument (Micro-Nano Sensing Technology Co., China), providing high temporal resolution for characterizing the dynamic response. Current–voltage (*I*–*V*) characteristics of the sensors were measured by using a Keithley 2450 source meter (Tektronix Inc., USA). The gas-sensing response (*R*_res_) was defined as *R*_res_ = *R*_a_/*R*_g_, where *R*_a_ and *R*_g_ represent the sensor’s resistance in air and the target gas, respectively. The response time (*T*_res_) is the interval from when the gas sensor is exposed to the target gas until the resistance change reaches 90% |*R*_a_ – *R*_g_|. Similarly, the recovery time (*T*_rec_) is the time interval from when the carrier gas purges the gas sensor until the resistance change returns to 90% |*R*_a_ – *R*_g_|.

## Results and Discussion

### Gas-Sensing Properties of SnO_2_-Ag

Gas responses of S0, SA1, SA2, and SA3 sensors toward 2000 ppm of CH_4_ at different temperatures are shown in Figure 1. We performed three repeated measurements for all tests. Error bars represent the standard deviation of the mean. Various samples with different thicknesses of the Ag layer exhibit different response amplitudes, with SA2 possessing the highest response across the entire measured temperature range. Figure 1a presents the original response curves of SA2 composite materials to 2000 ppm of CH_4_ at different temperatures. With increasing temperature, the responses increase, reach their peak values, and then decrease, indicating an optimal operating temperature at which the response is maximized. At the optimal operating temperature, oxygen adsorption and catalytic reactions with reducing gases are moderately enhanced. The response of different sensors to CH_4_ is summarized in Table S2 in the SI. As shown in Figure 1b–d, SA2 achieves optimal response/recovery times (19 s/21 s) at 350 °C, demonstrating a 50% improvement over pure SnO_2_ (i.e., S0) due to synergistic thermal-catalytic activation and optimized charge transfer at the SnO_2_-Ag interface. The response and recovery times of different sensors to CH_4_ can also be found in Table S3 in the SI.

**Figure 1:**
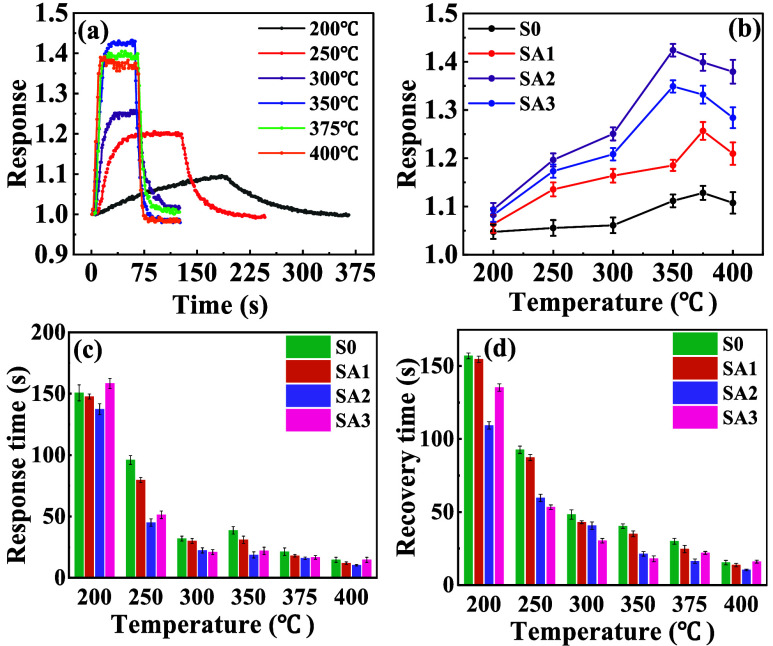
Response characteristics of different SA sensors to CH_4_. (a) Response curves of the SA2 sensor toward 2000 ppm CH_4_ at different operating temperatures. (b) Response of SA sensors toward 2000 ppm CH_4_ at different operating temperatures. (c) Response time of SA sensors toward 2000 ppm CH_4_ at different operating temperatures. (d) Recovery time of SA sensors toward 2000 ppm CH_4_ at different operating temperatures. The results in (b–d) are averaged from three independent measurements, and the error bars show the corresponding standard deviations.

The CH_4_ response initially increased and then decreased with higher Ag content, with SA2 (6 nm Ag) exhibiting the highest sensing response across the entire temperature range, achieving a response value of 1.42 to 2000 ppm of CH_4_. Therefore, 6 nm Ag, together with 350 °C, was selected as the optimal conditions for subsequent characterization with the introduction of ZnO in the sensing film. In addition, the responses of SnO_2_-Ag sensors to other combustible gases, such as CO and H_2_, were also characterized. Response curves, response amplitude, and response/recovery times of these sensors to CO and H_2_ are presented in Figure S1 and summarized in Table S2 in the SI. SA2 shows the largest response amplitude for both CO and H_2_ among all of the sensors. Furthermore, the optimal operating temperatures of SA2 for different target gases are at 225 °C for CO and 275 °C for H_2_, respectively. This difference enables effective discrimination among the three gases.

Doping Ag can reduce the operating temperature of gas sensing, which can be attributed to the catalytic role of Ag. Ag lowers the activation energy required for the reaction between CH_4_ molecules and adsorbed oxygen ions. However, if Ag concentration is low, it is likely to exist predominantly as isolated Ag^+^ within the SnO_2_ lattice, rather than in the form of metallic Ag nanoparticles with high catalytic activity.^30,31^ This ionic state exhibits limited effectiveness in lowering the activation energy for oxygen molecule dissociation. We can see from Figure 1b that the optimal operating temperature of SA3 does not show a decrease compared to that of pure SnO_2_ (S0). Considering that SA3 has the thinnest Ag layer (3 nm) among other samples, we infer that Ag dispersion is insufficient to establish a pervasive catalytic network across the sensor surface. Thus, the gas-sensing reactions are still primarily dominated by the noncatalyzed SnO_2_ surface.

### Gas-Sensing Properties of SnO_2_-Ag-ZnO

The gas responses of SA2, SAZ1, SAZ2, and SAZ3 sensors to 2000 ppm of CH_4_ at varying temperatures are illustrated in Figure 2a. Among these sensors, SAZ2 demonstrated the highest response magnitude across the entire tested temperature range. Notably, the ZnO thin-film coating exhibited no discernible influence on the temperature-dependent response characteristics of the sensor. The real-time response curves of gas sensors to varying CH_4_ concentrations at 350 °C are displayed in Figure 2b. The corresponding relationship between the gas concentration and sensor response is shown in Figure 2c. As the gas concentration increases, the response curves of the gas sensors to CH_4_ exhibit a distinct stepwise increase. The results demonstrate that depositing ZnO on the SA2 composite significantly enhances the CH_4_ sensing response. SAZ2 composite, incorporating a 10.86 nm-thick ZnO layer, exhibits the highest sensing response toward CH_4_ across all concentrations. Specifically, the response of SAZ2 to 2000 ppm of CH_4_ reaches 2.03, 1.79, and 1.43 times higher than those of S0 and SA2, respectively. SAZ2 composite exhibits enhanced sensing performance compared to SA2, likely due to the formation of an n-n heterojunction at their interfaces. This heterojunction promotes gas adsorption and reaction through interfacial effects, thereby enhancing the CH_4_ sensitivity. The SnO_2_-Ag-ZnO composite film sensors demonstrated rapid CH_4_ detection capabilities, achieving response and recovery times of 10 ± 1 and 8 ± 1.2 s, respectively, under 2000 ppm CH_4_ exposure, as shown in Figure 2d,e (cf. Table S3 in the SI). This enhanced kinetic performance is attributed to the magnetron-sputtered hierarchical structure, facilitating accelerated gas adsorption/desorption processes. Furthermore, SAZ2 demonstrates excellent reproducibility and stability in adsorption–desorption cycling measurements at 350 °C for 2000 ppm CH_4_ (see Figure S2a in the SI).

**Figure 2:**
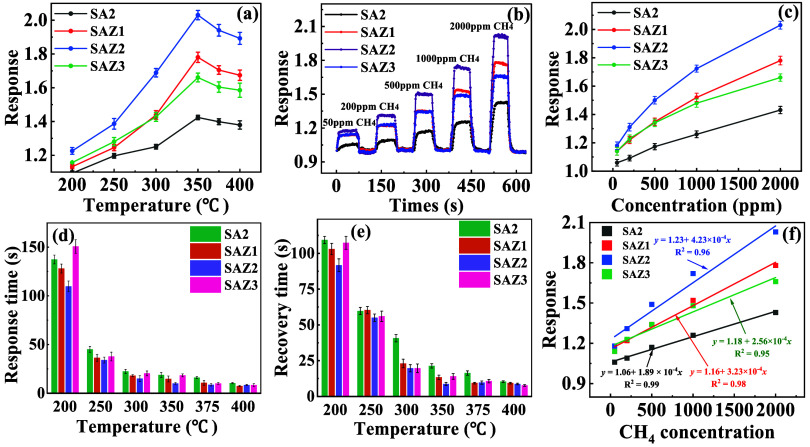
Response characteristics of different SAZ sensors to CH_4_. (a) Response of different SAZ sensors toward 2000 ppm CH_4_ at different operating temperatures. (b) Dynamic response curves of different SAZ sensors toward CH_4_ with concentrations ranging from 50 to 2000 ppm. (c) Response of different SAZ sensors toward 2000 ppm CH_4_ at 350 °C; (d) Response time of SAZ sensors toward 2000 ppm CH_4_ at different operating temperatures. (e) Recovery time of SAZ sensors toward 2000 ppm CH_4_ at different operating temperatures. (f) Determination of the limit of detection (LOD) by linear fitting the response as a function of CH_4_ concentration. The results in (a, c-e) are averaged from three independent measurements, and the error bars show the corresponding standard deviations.

We conducted additional experiments to evaluate the sensor’s response to potential interfering gases, including 20 ppm of nitrogen dioxide (NO_2_) and 20 ppm of ammonia (NH_3_). Four different sensors, SA2, SAZ1, SAZ2, and ZSA3, were operated at the optimal temperature of 350 °C for CH_4_ detection and exposed to five different gases, respectively: CH_4_ at 2000 ppm, H_2_ at 1000 ppm, and CO, NH_3_, and NO_2_ each at 20 ppm. These concentrations were chosen based on the hazard threshold levels of typical flammable gases.^32^ The corresponding responses were 2.03, 4.5, 1.15, 1.12, and 0.14, respectively (see Figure S2b in the SI). Although the response to H_2_ was higher than that to CH_4_, which can be attributed to the stronger reducing nature of H_2_, the two gases can still be clearly distinguished. With ZnO included in the compound sensing film, the response to CH_4_ increased significantly, while the response to NH_3_ remained almost unaffected. In addition, the response to NO_2_, an oxidizing gas, was much below 1. These distinct response patterns enable effective discrimination between CH_4_ and common interfering gases, proposing that a sensor array can be used to accurately distinguish the target gas and minimize environmental interference.

The limit of detection (LOD) is calculated using the formula LOD = 3­(*Std*/*m*), where *Std* is the standard deviation of 20 blank measurements reflecting the background noise level and *m* is the slope of the linear fitting equation. The linear fitting results between the sensor response and CH_4_ concentration are shown in Figure 2f. SAZ2 sensor exhibits a fitted relationship of *y* = 1.23 + 4.23 × 10^–4^ *x*, where *y* is the response value and *x* is the gas concentration, with a correlation coefficient (*R* ^2^) of 0.96, demonstrating excellent linearity across this concentration range. The LOD for SA2, SAZ1, SAZ2, and SAZ3 sensors are 43, 40, 33, and 42 ppm, respectively. Among them, the SAZ2 sensor shows the lowest detection limit, highlighting its strong potential for low-concentration CH_4_ detection applications.

To benchmark the sensing performance of our sensors, we selected typical examples of CH_4_ sensors fabricated by various methods for this comparison, as summarized in Table 2. Our sensors demonstrate substantial improvements compared to other reported CH_4_ sensors. They exhibit faster response and recovery times than those fabricated through hydrothermal synthesis, impregnation, and electrospinning techniques.^33,34^ Although the sensitivity improvement for CH_4_ remains moderate, the magnetron sputtering method employed for fabricating this MOS gas sensor offers distinct advantages, including the deposition of uniform and dense thin films over large-area substrates, enhanced crystallinity, reduced defect density, and excellent reproducibility. These characteristics make the technique particularly suitable for cost-effective mass production of gas sensors, ensuring both industrial scalability and minimized manufacturing costs.

**Table 2:** Benchmark of the Performance of the CH_4_ Sensor in This Work with Others

		Performance			
Material	Fabrication method	Concentration (ppm)	Response, *R*_a_/*R*_g_	*T*_resp_/*T*_recov_	refs
SnO_2_/rGO-Pt	hydrothermal synthesis	14,000	1.18	300 s/420 s	^33^
SnO_2_-Au	impregnation	500	8	347 s/260 s	^44^
SnO_2_/ZnO	hydrothermal synthesis	800	1.49	169 s/91 s	^28^
SnO_2_/ZnO-Pt	hydrothermal synthesis	800	5.08	142 s/87 s	^18^
SnO_2_-Pt	electrospun	1000	4.48	24.3 s/206.6 s	^34^
SnO_2_-Ag-ZnO	magnetron sputtering	2000	2.03	10 s/8 s	this work

### Material and Electrical Characterization

SEM analysis was performed on SA2 and SAZ samples to investigate the evolution of surface morphology with varying ZnO deposition levels, as shown in Figure 3. The surface topography of the SA2 sensor at different magnifications is presented in Figure S3 of the SI. The mismatch in thermal expansion coefficients between SnO_2_ (4.6 × 10–6 K^–1^)^35^ and Ag (18.9 × 10–6 K^–1^)^36^ induces internal stress during temperature fluctuations due to their differential expansion/contraction rates under constrained thin-film conditions. This stress manifests as surface cracks in the SA2 composite, as evidenced in Figure 3a. In the SAZ2 composite, well-dispersed ZnO particles form protruding spherical agglomerates, as shown in Figure 3c. This dome-shaped morphology enhances gas interaction by increasing the accessible surface area, enabling multidirectional gas molecule penetration and efficient binding with adsorption sites. Such structural optimization maximizes the utilization of metal-doped sensing sites, significantly improving gas-sensing response. In contrast, SAZ3 exhibits ZnO particle aggregation (Figure 3d), which reduces the effective gas-material contact area and diminishes the sensing performance, as observed in Figure 2b above.

**Figure 3:**
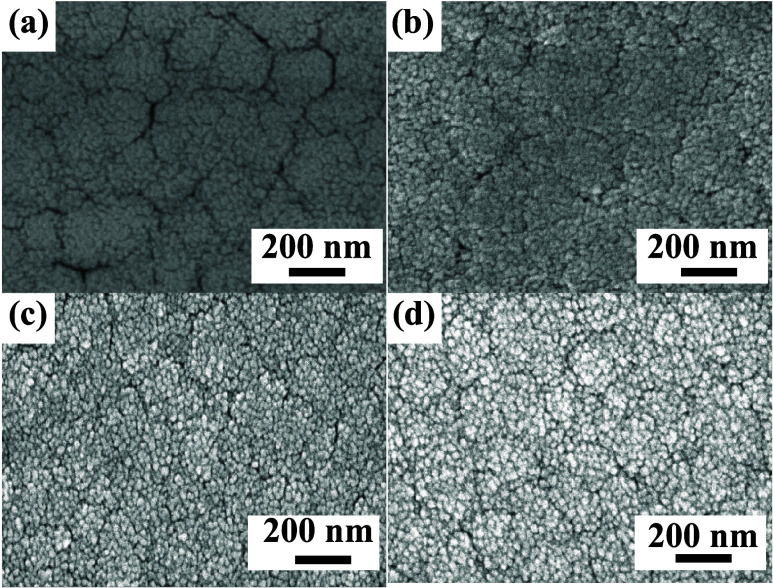
SEM images showing the surface morphology of different SAZ samples. (a) SA2; (b) SAZ1; (c) SAZ2; and (d) SAZ3.

The EDS spectra of various SnO_2_-based composites and the corresponding elemental mapping of SAZ2 can be found in Figure S4 in the SI. The EDS analysis reveals a homogeneous distribution of SnO_2_, Ag, and ZnO within the composite. Notably, the Sn content significantly exceeds that of Zn, with the spatial distribution of Zn aligning closely with that of Sn. This uniformity confirms the effective incorporation of metal and structural integration of the constituent phases, consistent with the material design objectives. The elemental mapping further validates the coexistence and spatial correlation of Sn, Ag, and Zn elements, demonstrating the successful fabrication of a multicomponent sensing architecture.

XPS characterization was performed to determine the elemental composition and chemical states of the sensor. High-resolution XPS spectra of O 1s, Ag 3d, Sn 3d, and Zn 2p of the SAZ samples and SA2 are displayed in Figure 4a–d, respectively. The O 1s spectrum can be deconvoluted into three peaks corresponding to distinct oxygen species (Figure 4a), i.e., lattice oxygen (O_L_) at ∼530 eV, oxygen vacancies (O_V_) at ∼532 eV, and chemisorbed oxygen (O_C_) at ∼533 eV.^37^ Notably, the increased O_V_ fraction after moderate ZnO doping suggests that controlled ZnO incorporation can enhance bandgap doping with O_v_. Ag peak^38^ in Figure 4b shifts toward higher energy for both peaks,^39^ which is due to the transition from AgO to Ag. The existence of a thicker ZnO layer will make it less likely that the underlying Ag will be oxidized. Sn 3d spectrum reveals a distinctive peak at 498.84 eV in SAZ2 in Figure 4c, significantly higher than the characteristic Sn^4+^ 3d_3/2_ binding energy in pristine SnO_2_-Ag, i.e., SA.^40^ The gradual attenuation of the Sn 3d_3/2_ and Sn 3d_5/2_ peak intensities with increasing ZnO content indicates a reduction in the SnO_2_ amount on the surface. Concurrently, the characteristic peak at 498.84 eV is attributed to the oxygen Auger transition (OKVV), confirming the altered surface electronic structure.^41^ It is worth noting that the intensity of Sn 3d peaks decreases with the increase of the thickness of ZnO, as shown in Figure 4c. The Zn 2p_1/2_ and Zn 2p_3/2_ signals are almost the same for all three SnO_2_-Ag-ZnO samples, indicating similar surface states of Zn element, as shown in Figure 4d.

**Figure 4:**
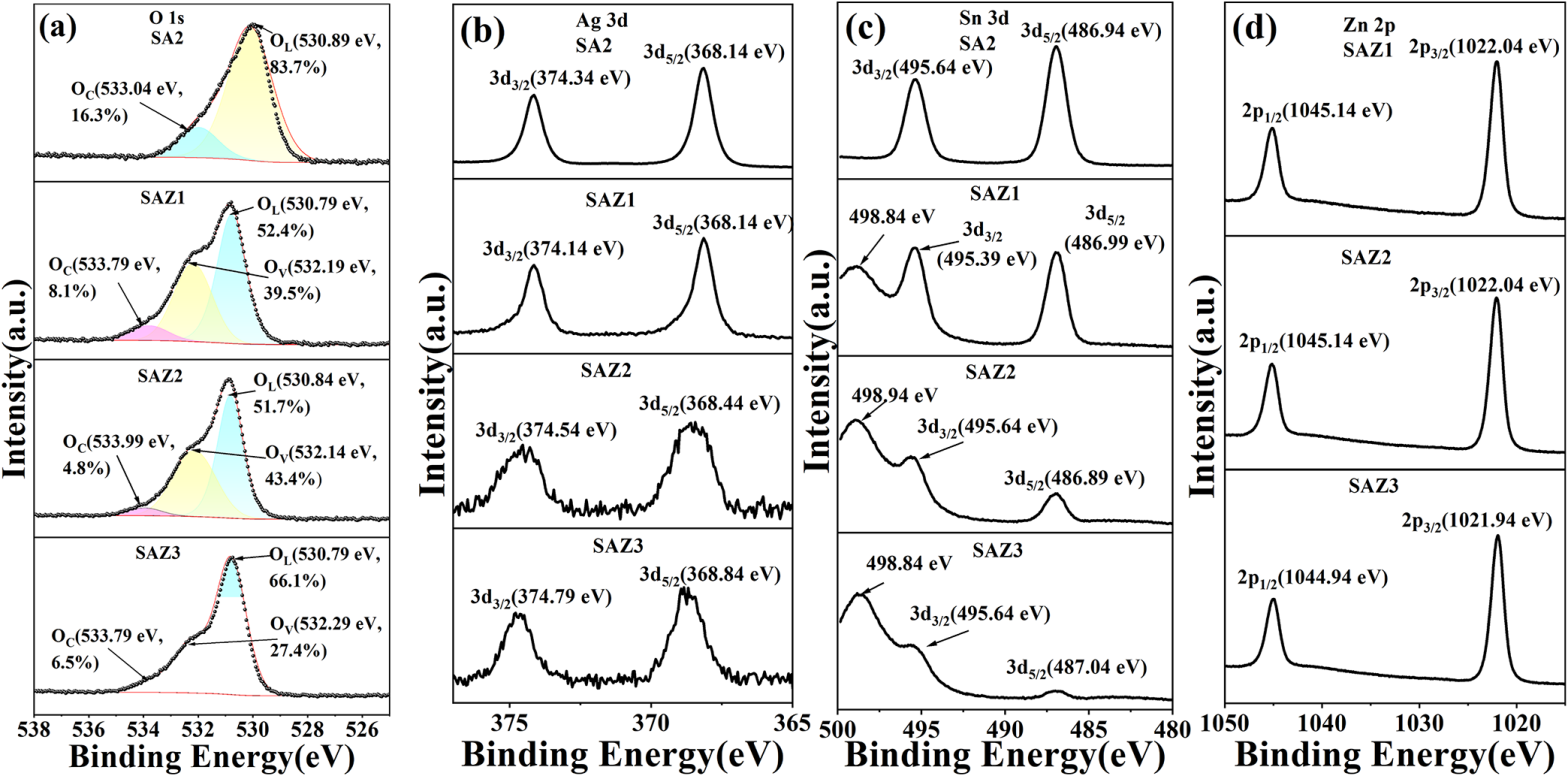
XPS spectra of SA2 and SAZ samples. (a) O 1s; (b) Ag 3d; (c) Sn 3d; and (d) Zn 2p.

The *I*–*V* characteristics of S0, SA2, and SAZ sensors under different temperatures under ambient conditions are compared in Figure 5. S0 exhibits linear *I*–*V* curves (Figure 5a), indicating the ohmic contact formation between Pt electrodes and the SnO_2_ sensing layer. In contrast, nonlinear *I*–*V* behavior is observed in SA2 across all tested temperatures, with the forward current exceeding the reverse current and distinct rectification behavior, as shown in Figure 5b. This asymmetry suggests the presence of a Schottky-like barrier at the metal-semiconductor interface. The difference in work function between SnO_2_ (4.6 eV) and Ag (4.72 eV) suggests the formation of a Schottky barrier at their interface, causing electrons to flow from SnO_2_ to Ag.^42,43^ This electron redistribution reduces the electron density in the depletion layer, resulting in a narrowed electron transport path and an overall increase in the electrical resistance of the sensing material. Thus, the thickening of the electron depletion layer on the SnO_2_ surface is a direct consequence of the interfacial charge transfer. The thickened depletion layer offers a larger reaction margin for reducing gases, such as CH_4_, resulting in a larger response amplitude for SA and SAZ sensors.^44^

**Figure 5:**
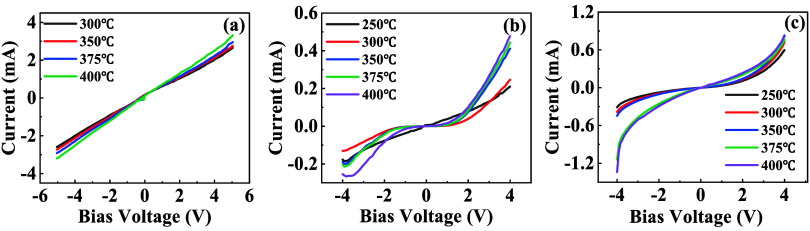
I-V curves of different SnO_2_ samples at different temperatures for (a) S0, (b) SA2, and (c) SAZ2.

An even more complicated nonlinear *I*–*V* curve is shown in SAZ2 with reversed polarity when temperatures exceed 375 °C; i.e., the reverse current exceeds the forward current. It implies forming heterojunction structures with thermally activated charge transport mechanisms. Due to the lower work function of SnO_2_ compared to that of ZnO (approximately 5.2 eV),^45^ electrons from the conduction band of the SnO_2_ film transfer to the conduction band of the ZnO film upon contact, until their Fermi levels align to achieve equilibrium. This charge redistribution results in the formation of an electron depletion layer at the SnO_2_ interface and a corresponding electron accumulation layer at the ZnO interface, thereby establishing a SnO_2_/ZnO heterojunction. When the SnO_2_/ZnO composite film is exposed to air, the SnO_2_ component, characterized by a higher concentration of oxygen vacancies, facilitates the adsorption of a greater amount of chemisorbed oxygen species (O_C_). This is also supported by XPS observation (Figure 5a). This mechanism is responsible for the enhanced gas-sensing performance observed in the composite material.

### Mixture Gas Classification

To comprehensively evaluate individual gas responses and cross-interference effects in complex atmospheres, a sensor array comprising four different CH_4_ sensors fabricated in this study (i.e., SA2, SAZ1, SAZ2, and SAZ3) was utilized. All measurements were conducted at 350 °C, encompassing three pure gases (CH_4_, CO, and H_2_) and multicomponent mixtures at varying concentrations. The initial gas concentrations used in this experiment were 20,000 ppm CH_4_, 199 ppm CO, and 20,100 ppm H_2_, balanced with N_2_. The gas mixtures were prepared by injecting specific volumes of individual gases into a gas bag using syringes, followed by a 24 h standing period to ensure complete homogenization prior to testing. The concentration ranges from 50 to 2000 ppm for CH_4_, from 0.2 to 20 ppm for CO, and from 10 to 200 ppm for H_2_. The total gas volume remained constant across all tests, while the volumetric ratios of the components were adjusted to achieve the desired mixture concentrations. The concentrations of each component in the gas mixture are listed in Table 3.

**Table 3:** Measured Concentrations of CH_4_ and Its Gas Mixtures

Class tag		1					2			
Gas concentration (ppm)	CH_4_	50	200	500	1000	2000	1600	1200	800	400
	CO	0	0	0	0	0	4	8	12	16
	H_2_	0	0	0	0	0	0	0	0	0
Class tag		3				4				
Gas concentration (ppm)	CH_4_	1600	1200	800	400	0	0	0	0	0
	CO	0	0	0	0	0.5	2	5	10	20
	H_2_	40	80	120	160	0	0	0	0	0
Class tag		5				6				
Gas concentration (ppm)	CH_4_	0	0	0	0	0	0	0	0	0
	CO	16	12	8	4	0	0	0	0	0
	H_2_	40	80	120	160	10	20	50	100	200
Class tag		7								
Gas concentration (ppm)	CH_4_	600	1200	400	400					
	CO	6	4	12	4					
	H_2_	600	400	400	1200					

The data set comprises response curves for 31 target gas mixtures, each measured five times, resulting in a total of 155 response curves. Each curve is recorded in 120 s with a sampling rate of 1 Hz, resulting in 120 sampling points. A spectrogram obtained by applying the Short-Time Fourier Transform (STFT) to each response curve reveals frequency domain information at different stages of the response. The STFT expands the one-dimensional signal into a two-dimensional representation. A Hanning window of 10 was used to segment the response curves. Considering a 1 Hz sampling frequency, the STFT spectra have a temporal resolution of 10 s. The overlap between adjacent windows was 50%. The length of the Fourier Transform was set to be 256.

Recent research on convolutional neural networks (CNNs) has focused on improving accuracy. Multiple small-scale CNNs often achieve a similar level of accuracy as a single large-scale CNN. However, small-scale CNNs offer apparent advantages: 1. They require less interserver communication during distributed training; 2. they are more suitable for deployment on hardware with limited memory. This study utilizes the SqueezeNet architecture, which organically integrates multiple small-scale CNNs. It is a well-known lightweight deep-learning model specifically designed for embedded applications.^46^ To date, few studies have reported the use of SqueezeNet for gas identification. *ImageNet* pretrained weights are utilized under a transfer learning framework, while preserving the original architecture (see Figure 6).^47^ The stacking configuration and parameters of the Fire modules can be found in Supporting Note 1 in the SI. The data set was randomly divided into the training set and test set with the ratio of 8:2. 5-fold cross-validation was used on the training set throughout the process of tuning model parameters and validation. The final model performance was tested on a test set. The model’s overall accuracy was determined by the average accuracy of the five trials.

**Figure 6:**
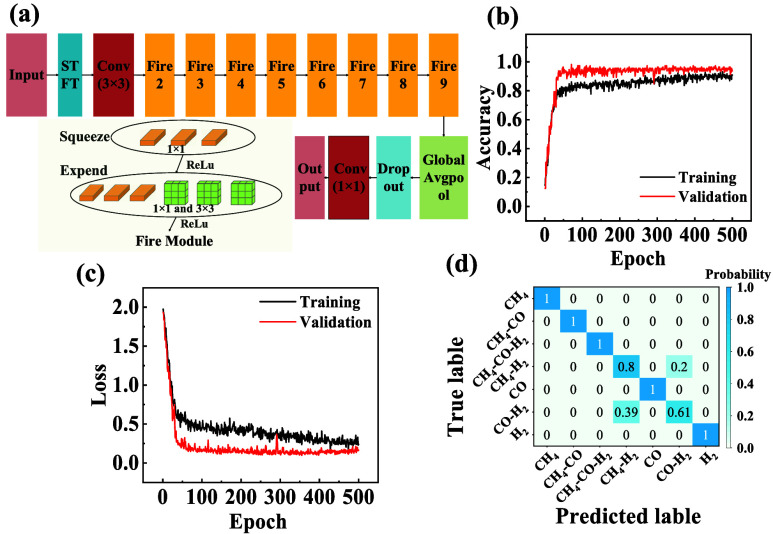
(a) Structure of the SqueezeNet model for gas classification. (b) Accuracy curves of the model during training and validation. (c) Loss curves of the model during training and validation. (d) Confusion matrix for gas classification of the test data set.

The model’s hyperparameters were optimized to improve accuracy. The batch size was set to 32, the learning rate was set to 0.0001, and the number of epochs was set to 500. The cross-entropy loss function was used as the evaluation criterion during model training, and the cross-entropy loss value reflects the difference between the model’s predictions and the actual labels. The training objective was to minimize this loss continuously. The Adam optimization algorithm was employed, which offers the advantages of simple implementation, high computational efficiency, low memory requirements, and the ability to adjust the learning rate adaptively during training. Dropout was incorporated into the model training to prevent overfitting and improve the generalization ability. The model exhibits near-perfect training accuracy (100%) but substantially lower validation accuracy (90.18%), indicating that it memorizes training-specific patterns rather than learning generalized features due to the lack of regularization (cf. Figure S5a,b in the SI).

This model demonstrates robust performance on the test data set, achieving an accuracy of 91.6%. The training dynamics and classification outcomes are listed in Figure 6b–d. The accuracy curves (Figure 6b) exhibit stable convergence across 500 training epochs. The training accuracy (the black curve) rises rapidly in the initial 50 epochs, reaching 85%, and then gradually plateaus at 91.6%, while the validation accuracy (the red curve) closely follows, indicating minimal overfitting. The final validation accuracy stabilizes at 91.6%, reflecting a strong generalization capability. This behavior is attributed to the integration of adaptive dropout layers and pretrained feature extraction, which regularizes the lightweight SqueezeNet backbone. The loss curves corroborate the stability of the training process (see Figure 6c). Both training and validation losses decrease monotonically, with the training loss converging to 0.18 and the validation loss converging to 0.22. The narrow gap between these curves further validates the effectiveness of the model’s regularization strategies, including early stopping and learning rate scheduling. The classification confusion matrix across seven gas categories is shown in Figure 6d. The model achieves a precision of over 95% for five major classes with minimal cross-class confusion. Notably, class 3 (CH_4_-H_2_) exhibits a slight misclassification rate toward class 5 (CO-H_2_), likely due to the spectral similarity in the STFT representations.

In addition, we varied the concentration ratios of the three gases, as shown in Table S4 and assessed the CH_4_ recognition accuracy under changing background levels of H_2_ and CO. The response signals were processed in the same way as previously described. Then the SqueezeNet model was trained, validated, and tested on the new data set. The results show that the SqueezeNet model maintains robust performance on the new data set, achieving an accuracy of 86.67% (See Figure S5 in the SI). The results demonstrate that the algorithm retains a strong selectivity advantage, with particularly high precision for high-concentration CH_4_ in environments containing low concentrations of H_2_ and CO.

The SqueezeNet architecture achieves remarkable efficiency, requiring only 0.22 million trainable parameters and occupying a storage space of merely 0.3–0.5 MB. The compressed network topology demonstrates exceptional parameter efficiency compared to conventional CNN architectures, notably achieving a 250× reduction in parameter count relative to AlexNet (60 million parameters) while maintaining competitive classification performance in terms of resource efficiency. By integrating pretrained feature extraction with adaptive dropout regularization, our model achieves robust classification accuracy without compromising computational sustainability. Such characteristics make it particularly suitable for deployment on resource-constrained embedded systems or edge devices, addressing the critical requirements of low-power operation, real-time responsiveness, and operational reliability in gas sensing applications.

## Conclusions

In this study, we successfully developed SnO_2_-Ag-ZnO ternary composite gas sensors with significantly enhanced sensitivity, selectivity, and stability for CH_4_ detection, achieved through material optimization. Ag doping effectively reduced the activation energy for CH_4_ oxidation, while the introduction of a ZnO heterostructure further boosted the sensing response by modulating the interfacial charge transfer. The sensors exhibited a response of 2 to 2000 ppm of CH_4_, with a reduced optimal operating temperature of 350 °C. Furthermore, the integration of a lightweight SqueezeNet deep-learning model enabled high-precision classification of CH_4_ and its gas mixtures, achieving 91.6% accuracy and effectively mitigating issues related to cross-interference in traditional sensors. The sensor fabrication process, compatible with magnetron sputtering, offers advantages such as low cost and high scalability, making it suitable for large-scale deployment in industrial processes such as coal mine safety monitoring. This work provides a novel and practical solution for high-accuracy CH_4_ detection in complex environments for a broad scope of real-world applications.

## Supplementary Material



## Notes

Response characteristics of SA sensors toward different gases at different temperatures; repeatability test of SAZ2 to 2000 ppm CH_4_ and selectivity of SAZ sensors; SEM images of SA2; EDS measurements of element composition of different SAZ sensors; model performance on the additional data set of ternary gas mixtures; materials and instruments used for preparation and characterization of sensors; comparison the responses of different sensors to CH_4_, CO, and H_2_; response and recovery times of the sensors to 2000 ppm CH_4_ at different temperatures; concentrations of CH_4_, H_2_, and CO in the ternary gas mixtures used in the experiments; SqueezeNet configuration (PDF)
