# Supplementary material for: Accurate Methane Detection in Combustible Gas Mixtures by Using SnO2‑Ag-ZnO Gas Sensors with Rapid Responses
Source: ACS Sens. 2025 Dec 15;11(1):290–8. doi: 10.1021/acssensors.5c02966 (PMC12836351; doi:10.1021/acssensors.5c02966)
Supplement: Supplementary file 1 [file se5c02966_si_001.pdf]

Supporting information

Accurate methane detection in combustible gas mixtures by using SnO_2-Ag-ZnO gas sensors with rapid responses

Mingzhi Jiao[1,2], Haojie Dong[1,2*], Yuting Qiao[1,2], Ruqi Guo[1,2], Chu Manh Hung[3], Nguyen Van Duy[3], Nguyen Duc Hoa[3], Chenyu Wen[4*]

1. National and Local Joint Engineering Laboratory of Internet Application Technology on Mine, China University of Mining and Technology, Xuzhou 221116, China
2. School of Information and Control Engineering, China University of Mining and Technology, Xuzhou 221116, China
3. International Training Institute of Materials Science, Hanoi University of Science and Technology, Hanoi 100000, Vietnam.
4. Division of Solid-State Electronics, Department of Electrical Engineering, Ångströmlaboratoriet, Uppsala University, Lägerhyddsvägen 1, Uppsala 75237, Sweden
* Corresponding authors: Haojie Dong: ts23060002a31@cumt.edu.cn; Chenyu Wen: chenyu.wen@angstrom.uu.se

Table of contents

Figure S1 Response characteristics of SA sensors toward different gases at different temperatures.
Figure S2 Repeatability test of SAZ2 to 2000 ppm CH_4 and selectivity of SAZ sensors.
Figure S3 SEM images of SA2 sensor at different magnifications.
Figure S4 EDS measurements of element composition of different SAZ sensors.
Figure S5 Model performance on the additional dataset of ternary gas mixtures shown in Table S4.

Table S1 Materials and instruments used for preparation and characterization of sensors.
Table S2 Comparison of the responses of different sensors to CH_4, CO, and H_2.
Table S3 Response/recovery time of the sensors to 2000 ppm CH_4 at different temperatures.
Table S4 Concentrations of CH_4, H_2, and CO in the ternary gas mixtures used in the experiments.
Table S5 The stacking configuration and parameters of the fire modules.

Supporting Note 1: SqueezeNet configuration.

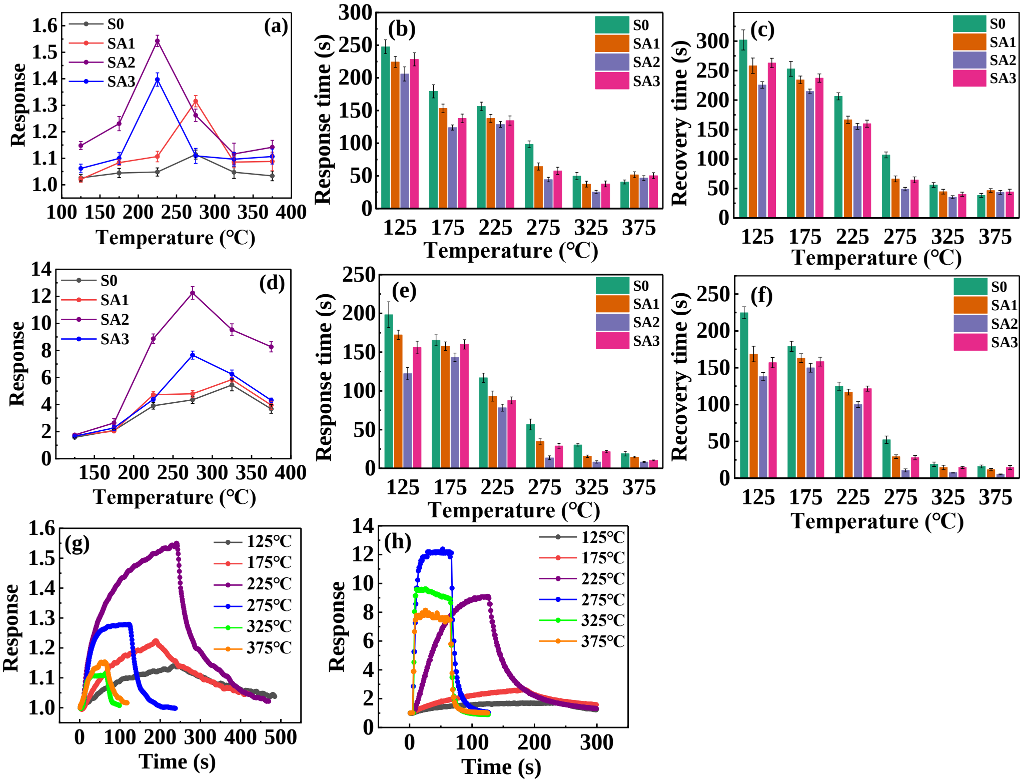

Figure S1. Response characteristics of SA sensors toward different gases at different temperatures. Response of SA sensors toward (a) 20 ppm CO and (d) 1000 ppm H_2 at different temperatures. Response time of SA sensors toward (b) 20 ppm CO and (e) 1000 ppm H_2 at different temperatures. Recovery time of SA sensors toward (c) 20 ppm CO and (f) 1000 ppm H_2 at different temperatures. Response curves of SA2 toward (g) 20 ppm CO and (h) 1000 ppm H_2 at different temperatures.

The responses of pure SnO_2 sensor (S0) and SA sensors to 20 ppm CO and 1000 ppm H_2 within the operating temperature range of 125–375 °C are illustrated in Fig. S1. As shown, introducing Ag into SnO_2 reduces the optimal operating temperature for CO from 275 °C (SA and SA1) to 225 °C (SA2 and SA3). The response curves of SA2 to 20 ppm CO (175–275 °C, Fig. S1g) and 1000 ppm H_2 (225–325 °C, Fig. S1h) clearly demonstrate its temperature-dependent gas-sensing characteristics. The specific responses of the three gases at various temperatures are summarized in Table S2. Compared with SA2, excessive Ag doping degrades the CO response to 1.39 for SA3. Similarly, for H_2 sensing, the optimum operating temperature decreases from 325 °C for SA and SA1 to 275 °C for SA2 and SA3. SA2 exhibits a significantly higher response of 12.13 to 1000 ppm H_2; however, further increasing the Ag content reduces this value to 7.69. As shown in Figs. S1b, c, e, and f, both the response and recovery times decrease with increasing temperature. At the optimal operating temperature of SA2 (225 °C), its response and recovery times to 20 ppm CO are 128.7 s and 155.3 s, respectively. For 1000 ppm H_2, SA2 shows response and

recovery times of 13.7 s and 10.7 s, respectively, at its optimal operating temperature of 275 °C.

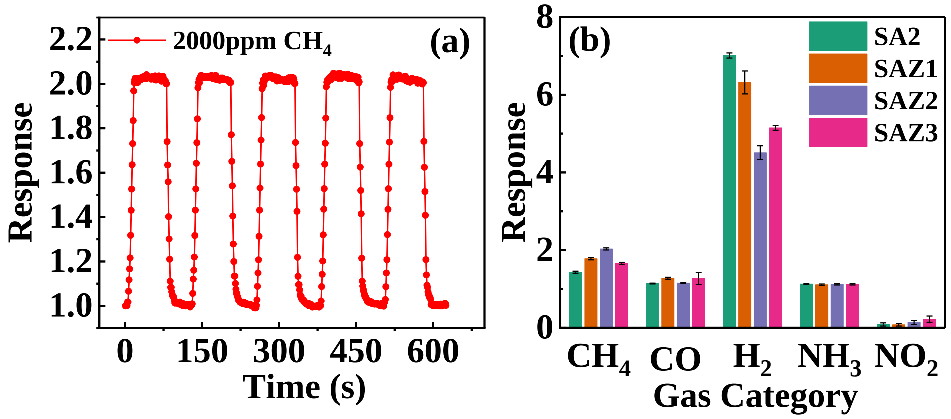

Figure S2. (a) Response curves of five adsorption-desorption cycles of SAZ2 to 2000 ppm CH_4 at 350°C. (b) Comparison of the response of SA sensors to different gases showing its selectivity. The results are averaged from three independent measurements, and the error bars show the corresponding standard deviation.

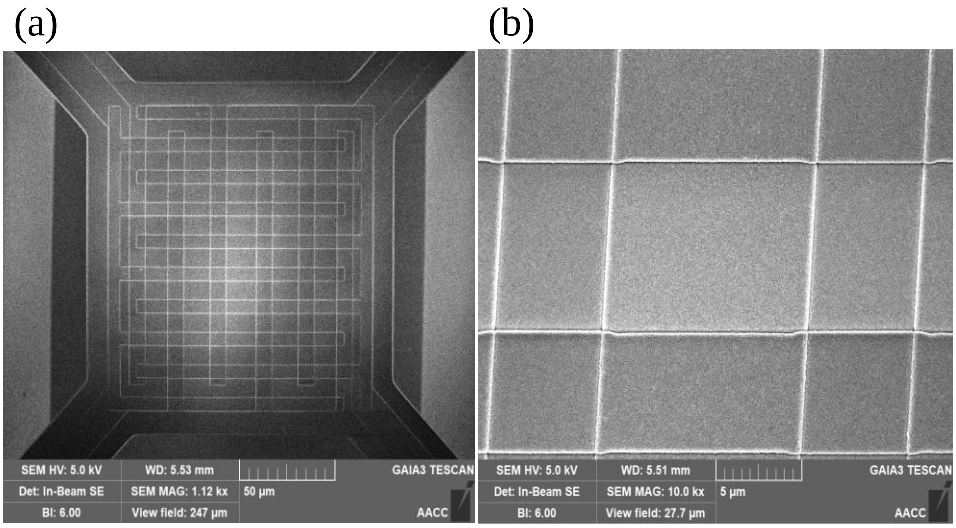

Figure S3. SEM images of SA2 sensor at different magnifications of (a) 1120 times and (b) 10k times.

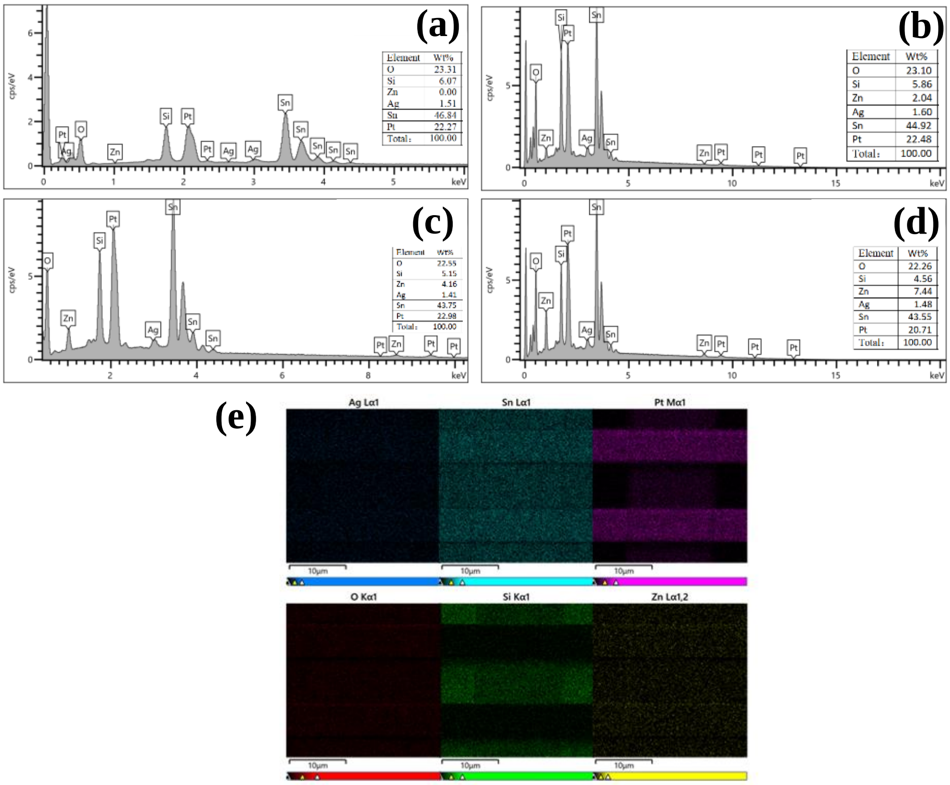

Figure S4. EDS measurements of element composition of different SAZ sensors. (a) SA2; (b) SAZ1; (c) SAZ2; (d) SAZ3. (e) EDS element mapping of SAZ2 sensor.

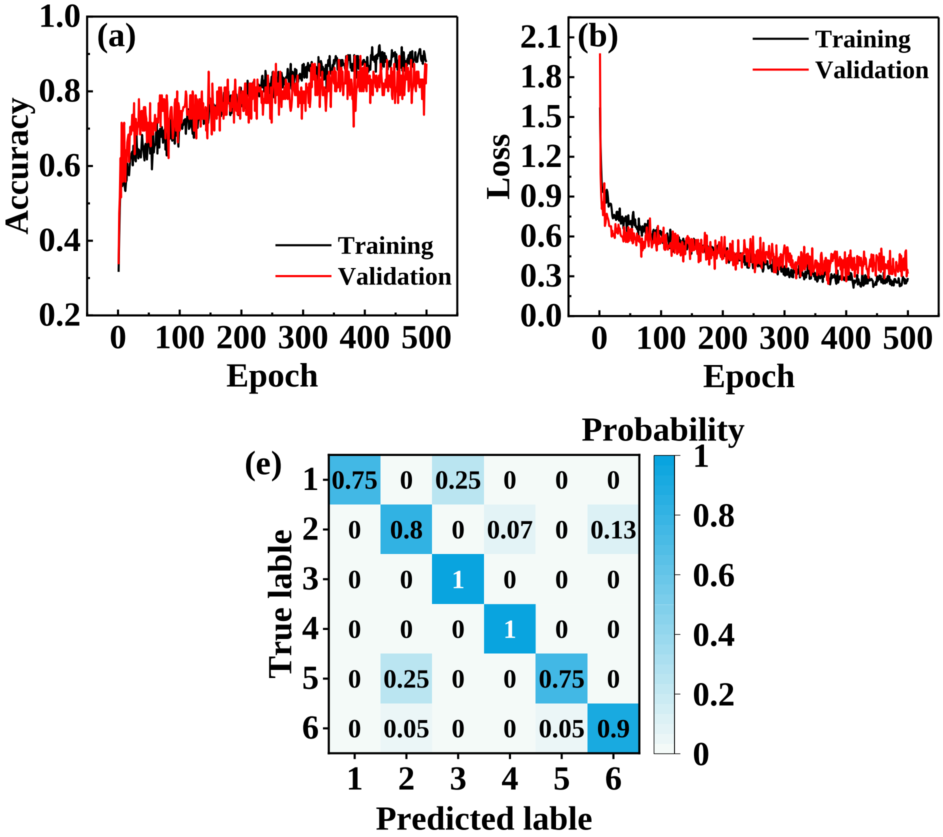

Figure S5. Model performance on the additional dataset of ternary gas mixtures shown in Table S4. (a) Accuracy curves of the model during training and validation. (b) Loss curves of the model during training and validation. (c) Confusion matrix for gas classification on the test dataset.

Table S1. Materials and instruments used for preparation and characterization of sensors

Materials/Instruments	Instrument type/Purity	Supplier
Scanning electron microscope	GAIA3	TESCAN, Czech Republic
Magnetron sputtering	MYDM-250	Hefei Qingya Electronics Co., China
Gas-sensitive test device	9064C	Micro-Nano Sensing Technology Co., China
Tin dioxide	Purity 99.99%	Zhongnuo Advanced Material Tech. Co., China
Zinc oxide	Purity 99.99%	Zhongnuo Advanced Material Tech. Co., China
Precious metal target silver	Purity 99.99%	Zhongnuo Advanced Material Tech. Co., China
Precious metal target gold	Purity 99.99%	Zhongnuo Advanced Material Tech. Co., China
Precious metal target palladium	Purity 99.99%	Zhongnuo Advanced Material Tech. Co., China

Table S2. Comparison of the responses of different sensors to CH_4, CO, and H_2

2000 ppm Methane							
Sensor	Temperature (°C)	200	250	300	350	375	400
S0	Response, R_a/R_g	1.04	1.05	1.06	1.10	1.13	1.11
SA1	Response, R_a/R_g	1.06	1.14	1.16	1.19	1.25	1.20
SA2	Response, R_a/R_g	1.09	1.19	1.25	1.42	1.39	1.38
SA3	Response, R_a/R_g	1.08	1.17	1.20	1.34	1.33	1.28
20 ppm Carbon monoxide							
Sensor	Temperature (°C)	125	175	225	275	325	375
S0	Response, R_a/R_g	1.03	1.04	1.05	1.11	1.04	1.03
SA1	Response, R_a/R_g	1.02	1.08	1.10	1.31	1.08	1.09
SA2	Response, R_a/R_g	1.14	1.23	1.53	1.27	1.12	1.14
SA3	Response, R_a/R_g	1.07	1.09	1.39	1.11	1.09	1.10
1000 ppm Hydrogen							
Sensor	Temperature (°C)	125	175	225	275	325	375
S0	Response, R_a/R_g	2.10	3.97	4.47	5.33	3.74	2.66
SA1	Response, R_a/R_g	2.04	4.71	4.83	5.84	3.93	3.07
SA2	Response, R_a/R_g	2.63	8.82	12.13	9.54	8.20	5.08
SA3	Response, R_a/R_g	2.32	4.37	7.69	6.27	4.13	2.94

Table S3. Response/recovery time of the sensors to 2000 ppm CH_4 at different temperatures

Response time (s)	S0	SA1	SA2	SA3	SAZ1	SAZ2	SAZ3
200℃	151	148	137	158	128	109.7	150.7
250℃	96	79.7	45	51.3	36.3	34	37.7
300℃	32	30	22.3	21	18	15	20.3
350℃	38.7	31	18.7	22	14.7	10	18.3
375℃	21.3	18	16	16.7	10.7	8.7	10
400℃	14.7	12	10.3	14.7	7.3	8.3	8.3
**Recovery time (s)**	**S0**	**SA1**	**SA2**	**SA3**	**SAZ1**	**SAZ2**	**SAZ3**
200℃	157	155	109	135	103	91.7	107.3
250℃	92.7	87.3	59.7	53.3	60.3	55	56
300℃	48.3	43	40.7	30.3	23	19.7	19.7
350℃	40.3	35	21.3	18	13.3	8.7	8.7
375℃	30	24.7	16.3	22	9.3	9.7	9. 7
400℃	15.3	13.7	10.3	16	9.3	8.7	8.7

Table S4. Concentrations of CH_4, H_2, and CO in the ternary gas mixtures used in the experiments

Class tag		1					2			
Gas concentration (ppm)	CH_4	50	200	500	1000	2000	600	1200	400	400
	CO	0	0	0	0	0	6	4	12	4
	H_2	0	0	0	0	0	600	400	400	1200
Class tag		3				4				
Gas concentration (ppm)	CH_4	1900	1900	1900	1900	0	0	0	0	0
	CO	0.2	0.4	0.6	0.8	0.5	2	5	10	20
	H_2	8	6	4	2	0	0	0	0	0
Class tag		5				6				
Gas concentration (ppm)	CH_4	100	100	100	100	0	0	0	0	0
	CO	3.8	7.6	11.4	15.2	0	0	0	0	0
	H_2	152	114	76	38	10	20	50	100	200

Supporting Note 1: SqueezeNet configuration

In SqueezeNet, the fire module serves as the core architectural component, engineered to reduce model parameter count while preserving representational capacity. The critical parameters (s1, e1, e3) in the fire module are defined as follows:

s1: Denotes the number of 1×1 convolutional filters in the squeeze layer, responsible for channel reduction through dimensionality compression.

e1: Specifies the number of 1×1 convolutional filters in the expand layer, enabling channel expansion while maintaining spatial resolution.

e3: Indicates the number of 3×3 convolutional filters in the expand layer, capturing spatially local patterns through larger receptive fields.

Table S5. The stacking configuration and parameters of the fire modules

Stage	Layer	Fire Module	Pooling operation
1	Conv	-	MaxPool (3×3,2)
2	Fire2	s1 = 16, e1 = 64, e3 = 64	None
3	Fire3	s1 = 16, e1 = 64, e3 = 64	MaxPool (3×3,2)
4	Fire4, Fire5	s1 = 32, e1 = 128, e3 = 128	None
5	Fire6, Fire7, Fire8	s1 = 48, e1 = 192, e3 = 192	MaxPool (3×3,2)
6	Fire9	s1 = 64, e1 = 256, e3 = 256	None

References

[S1] Jiao, MZ.; Chen, XY.; Hu, KX.; Qian, DY.; Zhao, XH.; Ding, EJ. Recent Developments in Nanomaterials-Based Conductive Methane Sensors. Rare Met. **2021**, *40*, 1515–1527.

[S2] Ning, X.; Yin, D.; Fan, Y.; Zhang, Q.; Du, P.; Zhang, D.; Chen, J.; & Lu, X. Plasmon-Enhanced Charge Separation and Surface Reactions Based on Ag-Loaded Transition-Metal Hydroxide for Photoelectrochemical Water Oxidation. Advanced Energy Materials, **2021**, *11*, No. 210040

[S3] Luo, X.; Jiang, R.; Ma, Z.; Yang, T.; Liu, H.; Deng, H.; Wu, W.; C. Dong, Du, X.-W. Regulating the Work Function of Silver Catalysts via Surface Engineering for Enhanced CO_2 Electroreduction. Phys. Chem. Chem. Phys, **2022**, *24*, No. 9188.

[S4] Li, Z.; Wang, R.; Xue, J.; Xing, X.; Yu, C.; Huang, T.; Chu, J.; Wang, K. L.; Dong, C.; Wei, Z.; Zhao, Y.; Wang, Z. K.; & Yang, Y. Core-Shell ZnO@SnO_2 Nanoparticles for Efficient Inorganic Perovskite Solar Cells. Journal of the American Chemical Society, **2019**, *141(44)*, 17610-17616.
